# Highly Efficient Flame Retardant Hybrid Composites Based on Calcium Alginate/Nano-Calcium Borate

**DOI:** 10.3390/polym10060625

**Published:** 2018-06-06

**Authors:** Zhenhui Liu, Zichao Li, Xihui Zhao, Lei Zhang, Qun Li

**Affiliations:** 1 College of Chemistry and Chemical Engineering, Qingdao University, Qingdao 266071, China; 2016020405@qdu.edu.cn (Zh.L.); zhaoxihui@qdu.edu.cn (X.Z.); 2017020832@qdu.edu.cn (L.Z.); 2 College of Life Sciences, Qingdao University, Qingdao 266071, China; zichaoli@qdu.edu.cn

**Keywords:** calcium alginate, nano-calcium borate, flame retardancy, pyrolysis, thermal degradation

## Abstract

Hybrid composites with low flammability based on renewable calcium alginate and nano-calcium borate were fabricated using an in situ method through a simple, eco-friendly vacuum drying process. The composites were characterized by X-ray diffractometry (XRD), Fourier transform infrared spectrum (FTIR), scanning electron microscopy (SEM), and thermogravimetric analysis (TGA). The combustion behavior and flammability of the composites were assessed by using the limiting oxygen index (LOI) and cone calorimetry (CONE) tests. The composites showed excellent thermal stability and achieved nonflammability with an LOI higher than 60. Pyrolysis was investigated using pyrolysis–gas chromatography–mass spectrometry (Py-GC-MS) and the results showed that fewer sorts of cracking products were produced from the hybrid composites compared with the calcium alginate. A possible thermal degradation mechanism of composites was proposed based on the experimental data. The combined results indicate that the calcium borate had a nano-effect, accumulating more freely in the hybrid composites and contributing significantly to both the solid phase and gas phase, resulting in an efficient improvement in the flame retardancy of the composites. Our study provides a novel material with promising potentiality for flame retardant applications.

## 1. Introduction

The energy crisis triggered by excessive consumption of fossil resources has inspired humans to search for chemicals and fuels which are sustainable and renewable [1]. Biomass represents the most abundant renewable carbon resource on Earth, and has attracted enormous attention from researchers in the recent decades [2,3]. Sodium alginate, as a natural polysaccharide, is an important class of biomass existing both in cell walls of brown algae and in soil bacteria as capsular saccharides [4,5]. Its characteristic structure consists of two random arranged uronic acids, β-d-mannuronic acid (M block), and α-l-guluronic acid (G block) [6,7,8]. The M and G possess different proportions and order depending on the source of alginic acid [9]. As the material is considered to be non-toxic, biocompatible, and biodegradable [10,11,12,13,14], it is widely used in biomedical materials for drug delivery and immobilization of microorganisms [15,16,17,18]. The G residues in alginate can gelate with divalent metal ions such as Ca^2+^ to form calcium alginate according to the popular egg-box model theory [19,20,21,22]. As calcium alginate has been reported as having limiting oxygen index (LOI) value of 48.0 and a peak heat release rate of 4.99 kW/m^2^ [23], it can be developed as a promising material with flame-retardant properties [24,25].

Nano-microparticle composites, with desirable physicochemical properties, high mechanical strength, and efficiently enhanced flame retardancy, have generated plenty of attention so far [26,27,28,29]. Liu reported that the 2CaO∙B_2_O_3_∙H_2_O nanomaterials [30] and Ca_2_B_2_O_5_∙H_2_O nanostructures [31] showed ideal flame-retardant properties with large specific surface area and low surface free energies. Lewin demonstrated that the nano-micropaticles in the composites can easily accumulate at the surface of combustion area and efficiently improve the stability of the polymers [32,33,34].

The aforementioned reports indicate that both the nano-calcium borate and calcium alginate show promise as low-flammability materials. To further improve the flame-retardant properties of calcium alginate, synergistic systems with other flame retardants were generally employed [11]. In this study, the uniform calcium alginate/nano-calcium borate composites were prepared through a simple and environmentally friendly method without usage of dispersant, through which the flame retardancy was improved by the nano-calcium borate composites as compared calcium alginate alone, and costs and time were also saved. To the knowledge of the authors, this is the first report on the preparation of hybrid composites using borate and sodium alginate as precursors via an in situ method, which is advantageous in comparison with traditional methods such as physical blends or chemical combination [35]. Furthermore, the improved flame-retardant properties and thermal stabilities of the prepared calcium alginate composites were tested, and the mechanism was proposed based on the experimental data.

## 2. Materials and Methods 

### 2.1. Materials

Sodium alginate was purchased from Guangfu Fine Chemical Research Institute (Tianjin, China). Calcium chloride and borax (sodium tetraborate decahydrate) were obtained from Kermel Chemical Reagent Co., Ltd. (Tianjin, China). All the other reagents were purchased from Sinopharm Chemical Reagent Co., Ltd. (Shanghai, China) and were of analytical grade.

### 2.2. Preparation of the Calcium Alginate/Nano-Calcium Borate Composites

**Scheme 1:**
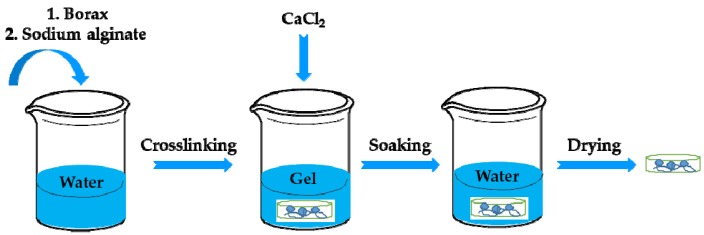
Preparation of calcium alginate/nano-calcium borate (CAB) composites.

The preparation process of the calcium alginate/calcium borate is shown schematically in Scheme 1. Borax (1.25 g) was blended with 250 mL distilled water. The solution was stirred at 70 °C for 5 min. Then, 10 g of sodium alginate was slowly added into borax solution with constant stirring. The obtained transparent gel was moved to glass utensil and immersed in 70% (*m*/*v*) calcium chloride solution after the gel shape was fixed. The pH of the solution was adjusted to 10 and kept at 30 °C for 10 h to ensure that the gel was cross-linked completely. Finally, the material was washed in distilled water, and then dried at 55 °C to obtain the calcium alginate/nano-calcium borate (CAB) composites. The preparation of calcium alginate (CA) was similar to that of CAB, while the use of borax was excluded in the process.

### 2.3. Measurements and Characterizations

#### 2.3.1. Limiting Oxygen Index (LOI)

The LOI tests were performed on a JF-3 type instrument (Jiangning Analytical Instrument Co., Nanjing, China) according to standard method ISO 4589-1:1996. Specimens of dimensions 100 mm × 40 mm × 2 mm were used for all the tests.

#### 2.3.2. Vertical Burning Rate (UL-94)

Vertical burning test was performed by using a CZF-2 vertical burning test instrument (Jiangning Analytical Instrument Co., Nanjing, China) according to ISO 5660 with sheet dimensions of 130 mm × 13 mm × 2 mm.

#### 2.3.3. X-ray Diffractometry (XRD)

XRD of the prepared samples and the cracking residues (heated in tube furnace with a heating rate 5 °C/min) was performed on an X-ray diffractometer (D/Max-RB, Rigaku Inc., Tokyo, Japan) equipped with Cu-Kα radiation at room temperature. The samples were scanned up to 90° in 2θ in a continuous mode.

#### 2.3.4. Fourier Transform Infrared (FTIR)

The chemical bonds in the samples and the cracking residues were determined by the FTIR spectroscopy (NICOLET iS50, Thermo Fisher Scientific Inc., Waltham, MA, USA) within the wave number range of 4500–500 cm^−1^.

#### 2.3.5. Scanning Electron Microscopy (SEM)

The morphology and microstructure of the samples and the residues were examined by a SEM (JSM 6390LV, JEOL Inc., Tokyo, Japan). All the sample surfaces studied were sprayed with gold to avoid charging under the electron beam.

#### 2.3.6. Thermogravimetric Analysis (TGA)

TGA was performed on a thermogravimetric analyzer (TG 209, NETZSCH Geraetebau GmbH, Selb, Germany) with a heating rate of 10 °C/min. The prepared 5-mg sample was examined with a flow rate of 50 mL/min from room temperature to 1000 °C in air and N_2_ atmosphere, respectively.

#### 2.3.7. Cone Calorimetry (CONE)

The combustion behavior of the samples was measured by a cone calorimeter device (M1354, Fire Testing Technology Ltd., East Grinstead, UK) according to ISO 5660, under an external heat flux of 50 kW/m^2^. All samples with dimensions 100 mm × 100 mm × 2 mm were wrapped in aluminum foil and placed in the horizontal position in a box with the same dimension.

#### 2.3.8. Pyrolysis–Gas Chromatograpgy–Mass Spectrometry (Py-GC-MS)

Py-GC-MS was performed using a fast pyrolysis analyzer (CDS 5200, CDS Analytical Inc., Oxford, MS, USA) coupled with a GC-MS (Clarus 680GC-SQ8MS, PerkinElmer, Inc., Shelton, CT, USA). The pyrolysis temperature was set at 200, 400, and 750 °C, and the carrier gas was He. The GC temperature program started at 50 °C and then increased to 300 °C within 15 min. The product of the compounds can be identified by the characterized GC/MS spectrums, according to comparison with the National Institute of Standards and Technology (NIST) library and other reports.

## 3. Results and Discussion

### 3.1. LOI and UL-94

The flame retardancy of CAB and CA was estimated by LOI and UL-94 vertical burning tests, and the results are listed in Table 1. It can be seen that both CAB and CA exhibited notable flame-retardant properties with high LOI values. It can be seen that both CAB and CA cannot be ignited and exhibit notable flame-retardant properties with high LOI values. Moreover, it can be noted that the LOI was increased from 48 (CA) to over 60 (CAB) by the introduction of 4% borax, indicating the important contribution of calcium borate to the high flame-retardant efficiency of CAB.

### 3.2. XRD Analysis

**Figure 1:**
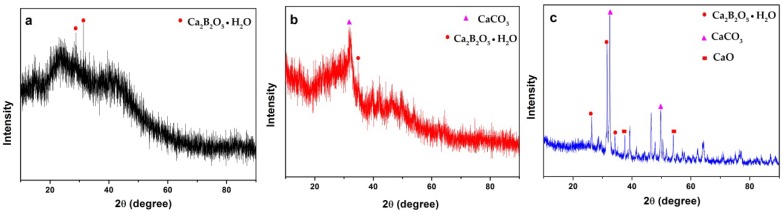
XRD patterns of (**a**) unheated CAB; (**b**) CAB heated to 350 °C; (**c**) CAB heated to 700 °C.

Figure 1 shows the XRD patterns of CAB and the cracking residues after heating at 350 and 700 °C. It can be noted that CAB was a kind of polymer material displaying crystallinity. The diffraction peaks of 28.46° and 31.34° seen in Figure 1a can be assigned to Ca_2_B_2_O_5_∙H_2_O (JCPDS PDF# 22-0139), suggesting CAB was synthesized. The new peaks of 32.96° can be observed from Figure 1b, indicating the formation of calcium carbonate (JCPDS PDF# 33-0268). As shown in Figure 1c, the peaks of 37.41° and 53.93° can be attributed to the decomposition of calcium carbonate into calcium oxide (JCPDS PDF# 82-1690) when CAB was further heated to 700 °C. Moreover, the existence of the calcium borate in the composites can be noted even at the higher heating temperature.

### 3.3. FTIR Analysis

**Figure 2:**
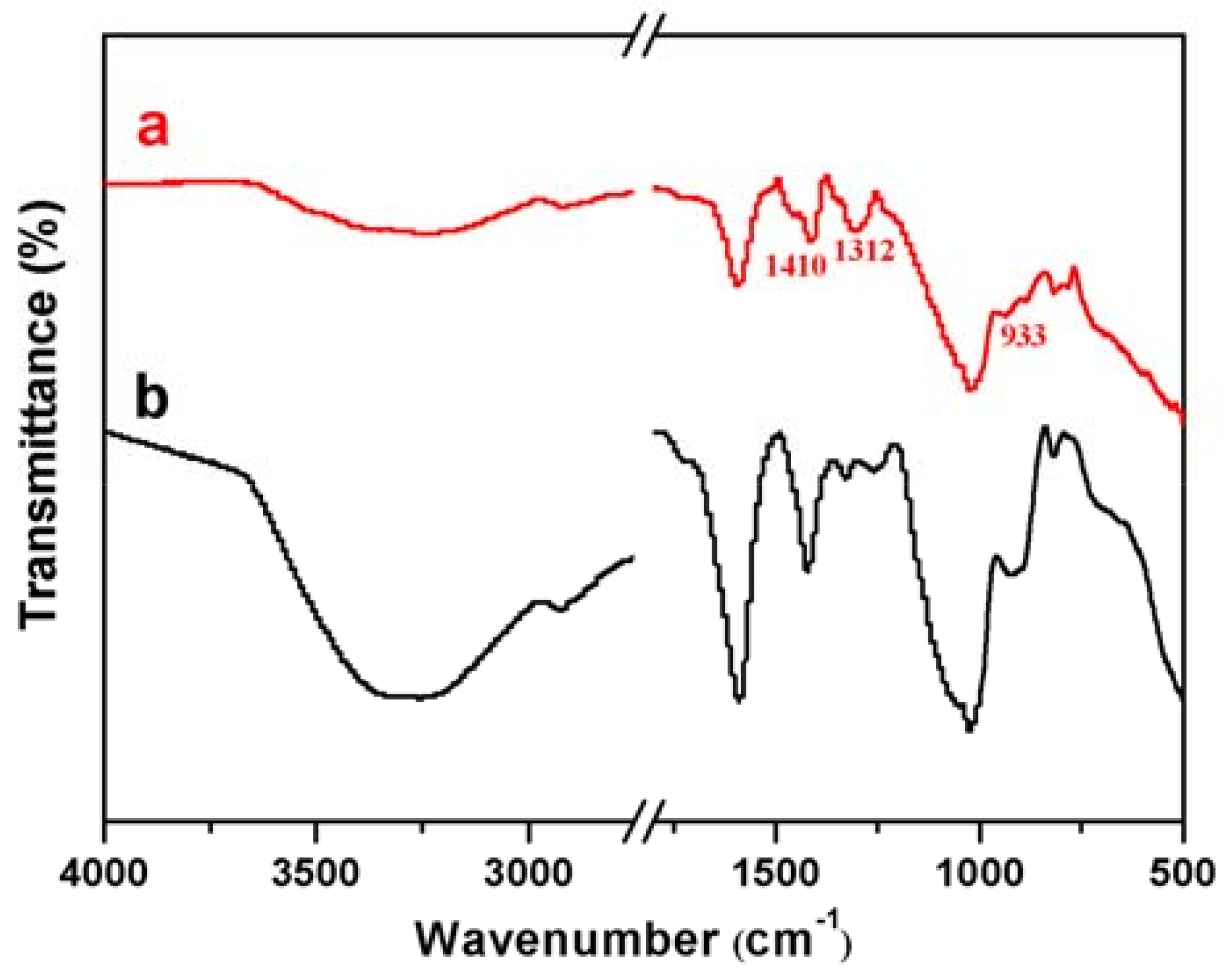
FTIR spectra of (a) CAB and (b) CA.

As seen in the FTIR spectra of the samples shown in Figure 2, a series of characteristic absorption peaks for CA were observed at 3285 cm^−1^ (O–H stretching vibration), 2915 cm^−1^ (–CH_2_), 1584 cm^−1^ (C=O stretching vibration) [36], 1253 cm^−1^ and 1429 cm^−1^ (assigned to the symmetric and asymmetric vibrations of –COO), 1331 cm^−1^ (–OH deformation vibration) [37], 1029 and 914 cm^−1^ (attributed to the symmetric and asymmetric vibrations of C–O–C), and 816 cm^−1^ (–CH_2_ in plane sway vibration), respectively. Noticeably, the peak at 933 cm^−1^ can be assigned to the B–O symmetric stretching, while the two peaks with similar intensities and shapes appearing at 1312 cm^−1^ and 1410 cm^−1^ can be ascribed to the asymmetric stretching of B–O, indicating the formation of calcium borate in CAB, although most of other chemical groups were detected in both of the samples.

### 3.4. SEM

**Figure 3:**
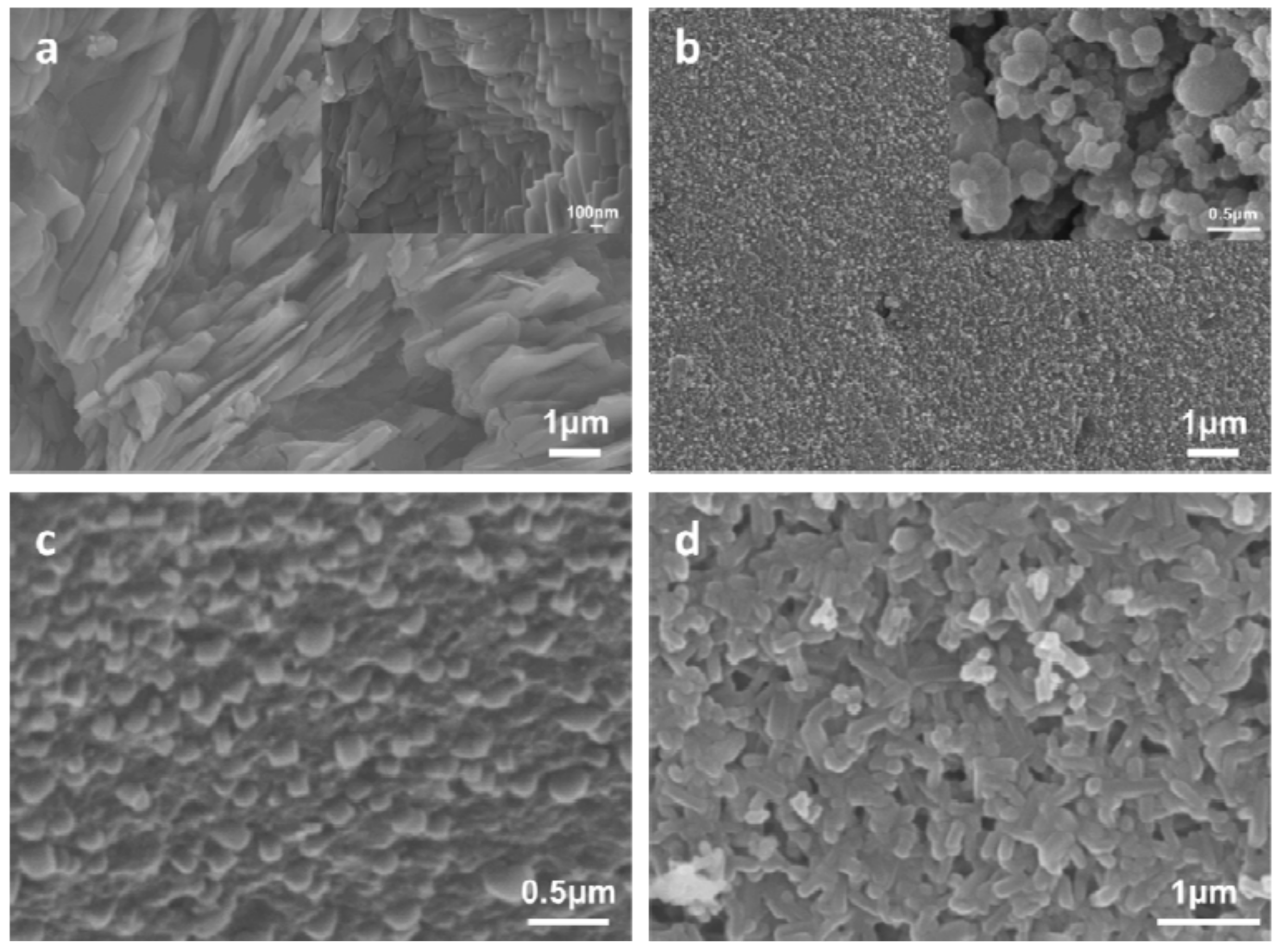
SEM images of (**a**) CA; (**b**) CAB; (**c**) CAB heated at 500 °C; and (**d**) CAB heated at 700 °C. The insets in (**a**,**b**) are the magnified images, respectively.

The SEM images of the prepared samples and the cracking residues of CAB after combustion at different temperatures are shown in Figure 3. As seen in Figure 3a, the surface with schist-like shapes of about 100-nm thickness can be observed in CA. It can be observed from Figure 3b that the calcium borate was uniformly embedded in CA, and the nano-calcium borate particles were self-assembled into sphere shapes with the size of around 100–200 nm, as displayed in the inset of Figure 3b. As shown in Figure 3c, the nano-calcium borate particles kept their original morphology and no notable change can be observed after the CAB was heated to 500 °C. However, as seen in Figure 3d, the particles at the surface of CAB were changed to worm-like shapes after being heated to 700 °C, which may be a result of the calcium borate melting and loss of the modification of CA at the higher temperature.

### 3.5. TGA and DTG

#### 3.5.1. Thermal-Oxidative Degradation Behaviors under Air

**Figure 4:**
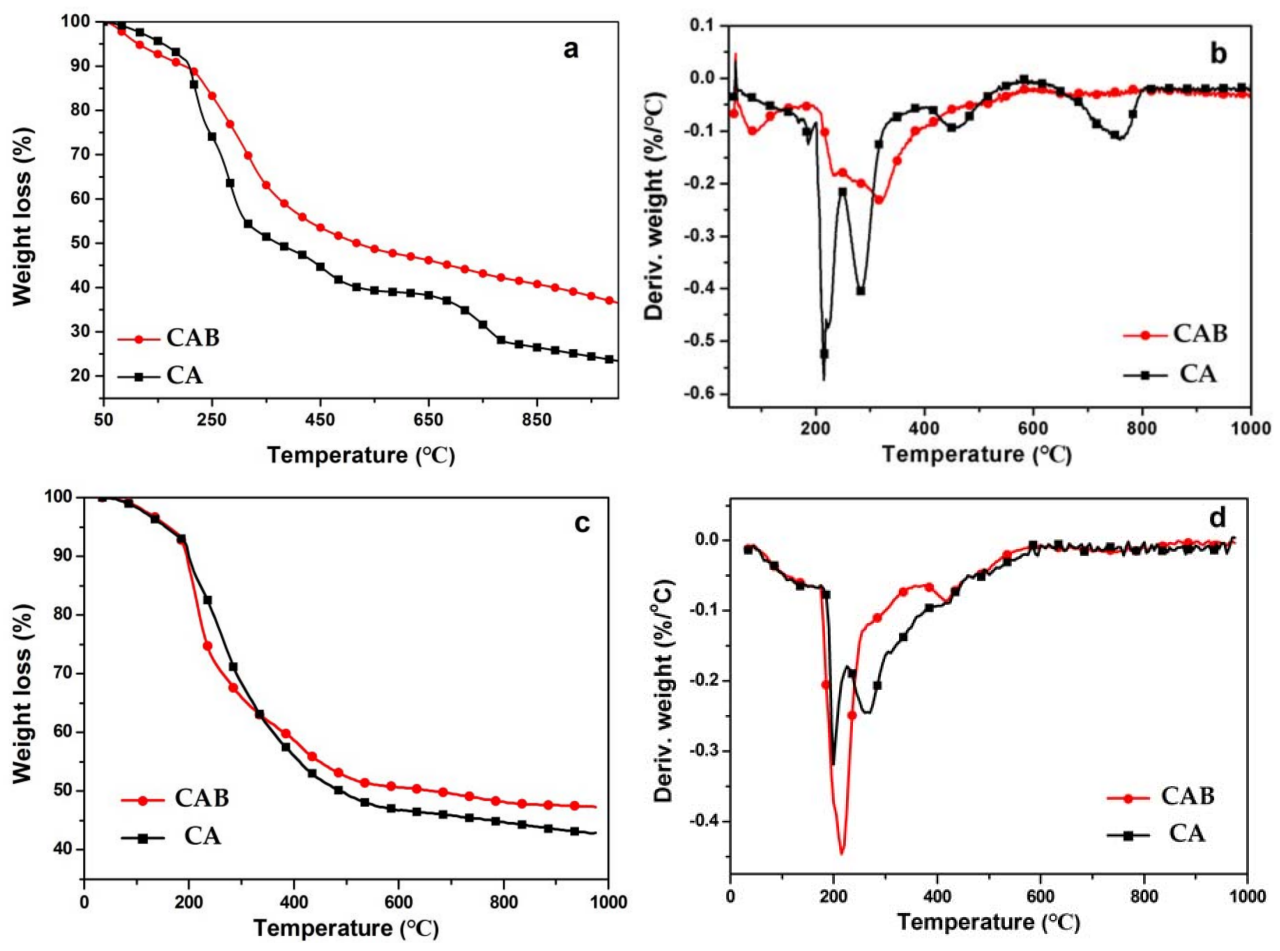
(**a**) TGA and (**b**) DTG curves of CAB and CA under air; (**c**) TGA and (**d**) DTG curves of CAB and CA under N_2_.

As seen in Figure 4a,b, CAB and CA exhibited similar degradation trends, with certain differences. It can be observed that the first step occurred at temperatures ranging from 50 to 230 °C, corresponding to the water desorption and for the CAB including the loss of crystal water in the calcium borate. The main degradation of CAB and CA occurred at 230–450 °C, which can be mainly associated with the destruction of glycosidic bonds. Moreover, the performance of CAB and CA at 450–700 °C can be ascribed to the oxidation of the previously formed carbonaceous char materials. The results indicated that CAB generated more residues compared with CA, suggesting that CAB possessed better thermal-oxidative stability.

#### 3.5.2. Thermal Degradation Behaviors under N_2_

The TG curves of CAB and CA in N_2_ are displayed in Figure 4c,d, and two main stages were found based on the patterns. An about 7% weight loss of CAB and CA can be observed from the first stage, which may be due to the moisture evaporation from ambient temperature to 200 °C. Over 200 °C, the main weight losses for CAB and CA were observed, which can be associated with the fracture of the glycosidic bonds, and dehydration, decarboxylation, and decarbonylation of alginate. Furthermore, CAB showed higher weight loss than that of CA at the range of 200–350 °C, which may be ascribed to the loss of crystal water in the calcium borate. In addition, the maximum-rate decomposition temperature of CAB (410 °C) was higher than that of CA (275 °C) in the second stage. This result may be in the reason that the calcium borate in the composites was melted and then protected the alginate from decomposition.

In addition, it seems borate can act as an endothermic cooling agent by losing its own crystal water in the gas phase at temperatures ranging from 200 to 350 °C, while it melted and formed a glassy cover at the surface to shelter the material from the heat in the solid phase at the higher temperature. This indicates that the introduction of calcium borate can improve the thermal stability of CA.

TGA was performed in air and N_2_, respectively, to understand the effect of oxygen on the degradation for CAB at high temperature by comparison. The TG curve of CAB in air was in accordance with that in N_2_ below 200 °C, indicating that the oxygen did not take part in the degradation process of CAB until the temperature reached 200 °C. It can be noted that CAB degraded much more in air than in N_2_, which led to the amount of the residues for CAB in air was less than that in N_2_. The residues were relatively stable once formed under both of the atmosphere.

### 3.6. CONE

**Figure 5:**
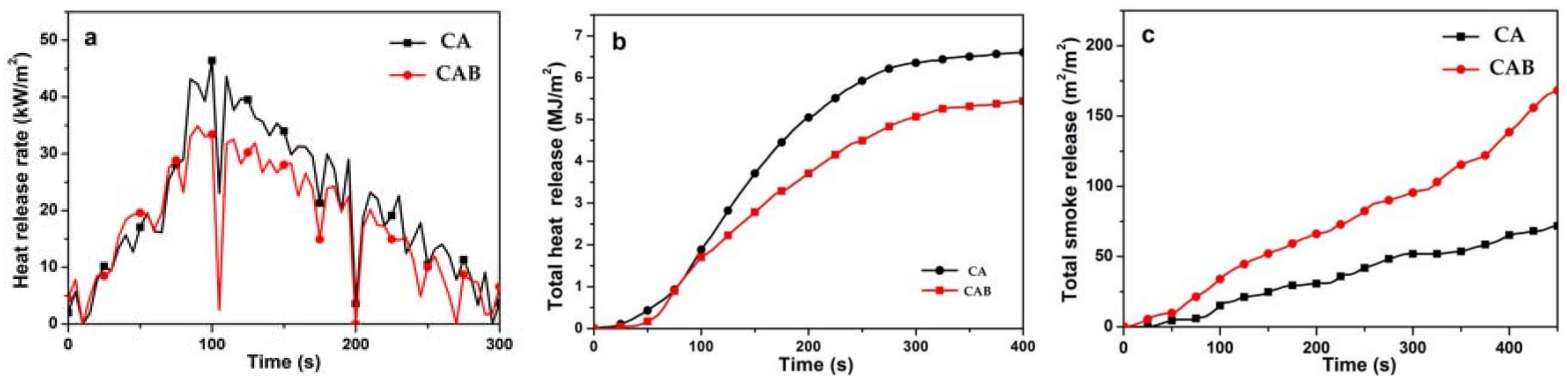
(**a**) HRR, (**b**) THR, and (**c**) TSR curves of CA and CAB changing with time.

CONE is one of the most effective methods to evaluate the flame-retardant properties of the tested materials through simulating fire conditions in the real world [38,39]. It was used to study the combustion behavior of CAB and CA, as displayed in Figure 5 with related data listed in Table 2. The heat release rate (HRR) is an important parameter to characterize the fire safety and a lower HRR value indicates slower spread of fire [34]. It can be seen from Figure 5a that the HRR of CAB was much lower than that of CA, while CAB showed a decreased peak heat release rate (PHRR) value compared to that of CA. It can be observed from Figure 5b that the total heat release (THR) of the two samples increased over time. CAB showed lower values of HRR and THR, indicating it possessed better flame-retardant properties compared to CA. This may be attributed to that the calcium borate in CAB produced a residue layer and acted as an insulating barrier between the fire and the material during combustion. As shown in Figure 5c, CAB produced more smoke than CA did, which may be ascribed to that the surface of CA was covered by calcium borate in the composites which could not be completely burned under the condition that the oxygen was partially insulated. Moreover, the residual amount of CAB (35.3%) was higher than that of CA (33.1%) at 360 s. Combining the above-mentioned results, CAB showed better flame-retardant properties than CA in the CONE tests.

### 3.7. Py-GC-MS

**Figure 6:**
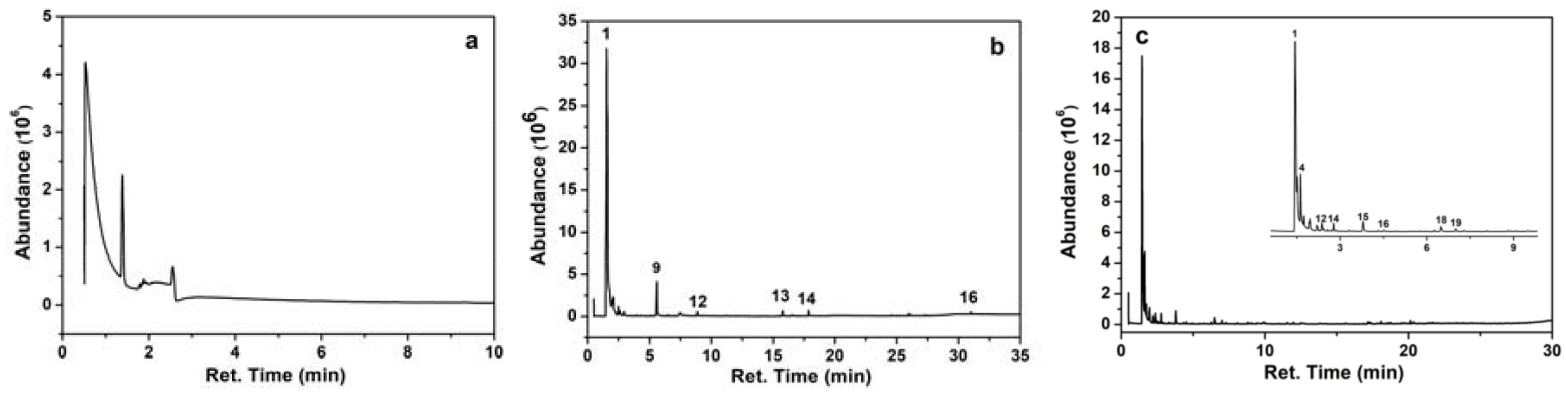
Py-GC-MS spectra of CAB at 200 °C (**a**), 400 °C (**b**), and 750 °C (**c**). The inset in (**c**) is the magnified spectrum of retention time within 9.5 min.

The Py-GC-MS analysis was conducted to research the main components of gaseous products of CAB in the pyrolysis process [40]. Most of the produced compounds were identified by comparison with the NIST library and listed in Table 3. Figure 6 shows the chromatograms of CAB obtained from Py-GC-MS tested at 200, 400, and 750 °C, respectively. The main compounds generated at 400 °C were as follows: carbon dioxide, 2,3,4,5-tetrahydroxyvaleric acid, dimethyl ether, 2-furaldehyde, and 5-methyl-furfural. It can be noted that the main products can be divided into four groups including carbon dioxide, organic acid (i.e., acetic acid), ketone (i.e., 2-propanone and 3-hydroxy-2-butanone), and aldehyde (i.e., acetaldehyde). Compared with the pyrolysis compounds of CA, much more ketone and aldehyde were produced in the decomposition compounds of CAB [23]. It can be seen that the decomposition products of CAB at 750 °C were mainly aromatic hydrocarbons, alcohol, and olefin which was further decomposed, suggesting that the introduction of calcium borate exerted positive effects on the degradation process of CA, and thus made it more resistant to the high temperature.

**Figure 7:**
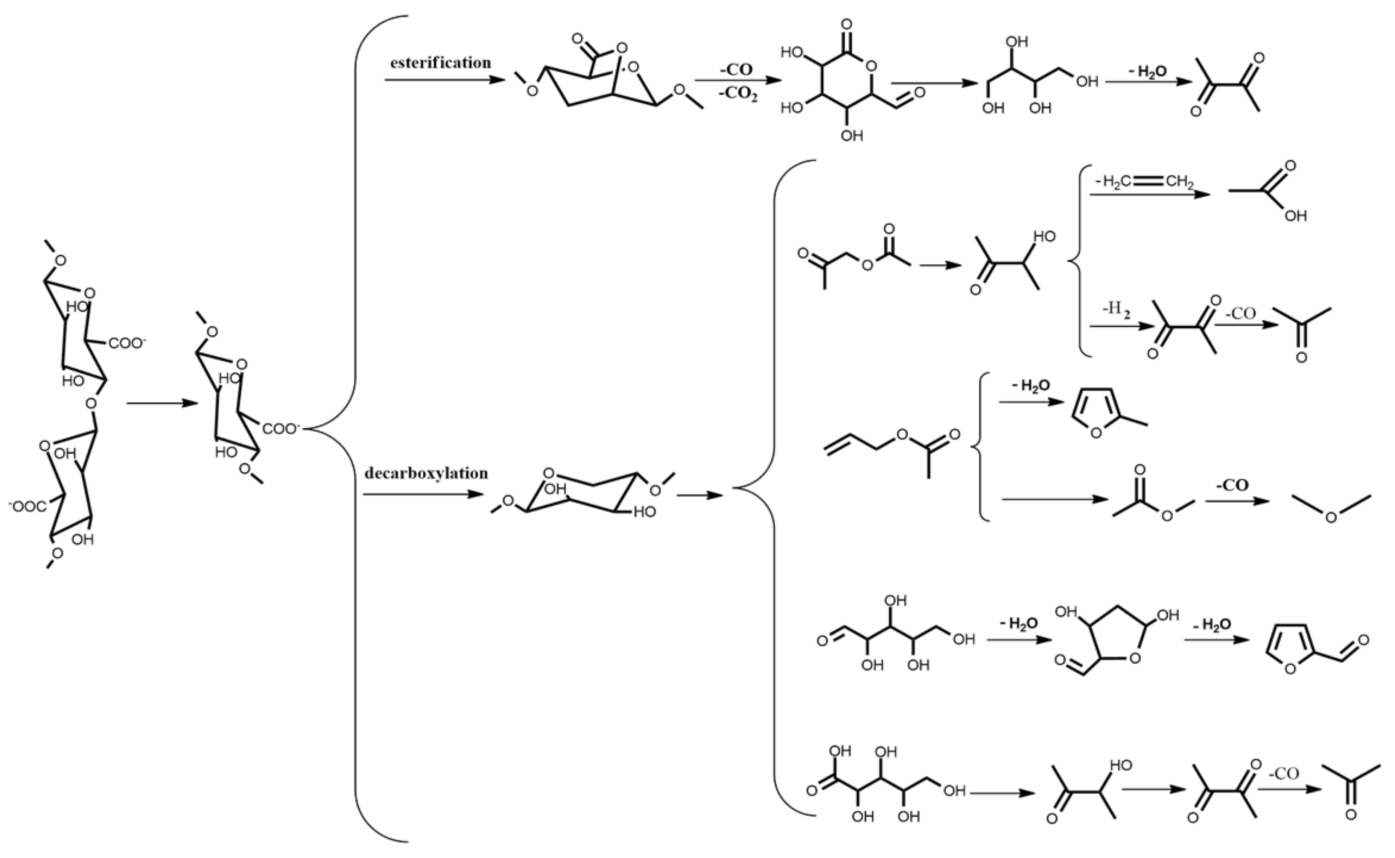
Proposed pyrolysis mechanism of CAB.

According to the analysis of Py-GC-MS, the proposed thermal degradation mechanism of CAB is shown in Figure 7. The main chain of CA was broken to produce G and M monomers at 400 °C. The monomer was firstly esterified by releasing H_2_O, followed by the ring-opening reaction of the intermediate products while losing some small molecules such as H_2_O, CO, etc., and lastly 2-propanone was formed in the pathway. On the other hand, the monomer was decarboxylated by releasing CO_2_. Subsequently, the reaction occurred as follows: (1) 2-furaldehyde was produced through tautomerization and cyclic hemiacetal and dehydration reactions after the cyclic fragmentation of 2,3,4,5-tetrahydroxypentanal; (2) 2,3-butanedione and acetic acid was generated by the loss of H_2_ and ethylene to 3-hydroxybutanone produced through the acetone acetate releasing CO; and (3) 2-methylfuran and dimethyl ether was formed by vinyl propionate losing a series of small molecules.

## 4. Conclusions

A type of hybrid composites based on calcium alginate and nano-borate particles was prepared by an in situ and post-cross-linking method, which was simple, eco-friendly, and economical. The combustion results indicated that the prepared composites possessed better thermal stability and flame-retardant properties. The inorganic nanoparticles can further suppress the flammability of the calcium alginate. Both nano-calcium borate and calcium alginate played crucial roles in improving the flame retardancy of CAB during combustion. The flame-retardant mechanism was proposed based on the experimental data, in which the calcium borate can cover the surface of calcium alginate, resulting in the decrease in the flame spread rate by forming barriers to prevent heat, oxygen, and smoke particles. The prepared materials show enormous potential to be applied in the building insulation materials and textile industry.

## Figures and Tables

**Table 1:** Combustion parameters obtained using the limiting oxygen index (LOI) and vertical burning rate (UL-94). CA: calcium alginate.

Samples	LOI (%)	UL-94
CA	48	V-0
CAB	>60	V-0

**Table 2:** Combustion parameters obtained from cone calorimetry (CONE). PHRR: peak heat release rate; THR: total heat release; TSR: total smoke release.

Samples	THR (MJ/m^2^)	PHRR (kW/m^2^)	TSR (m^2^/m^2^)	Residue (%)
CA	6.76	46.6	70.5	33.1
CAB	5.51	34.3	130.1	35.3

**Table 3:** Pyrolysis products of the CAB at different temperatures.

Molecular Structure Name of Compound		*T* = 250 °C		*T* = 400 °C		*T* = 750 °C	
		Time	Area	Time	Area	Time	Area
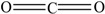	Carbon dioxide	0.53	89.27	2.21	0.78	1.44	51.26
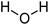	Water	1.39	7.70	-	-	-	-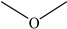	Dimethyl ether	-	-	1.73	7.96	-	-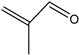	2-Propenal	-	-	2.36	0.27	-	-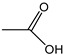	Acetic acid	-	-	2.49	0.84	-	-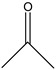	2-Propanone	-	-	2.63	0.41	1.63	11.97
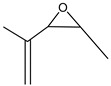	2-Oxiranecarboxylic-3-methyl acid	-	-	2.87	0.16	-	-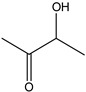	2-Butanone-3-hydroxy	-	-	2.94	0.41	-	-
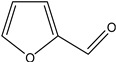	2-Furaldehyde	-	-	5.59	5.78	-	-
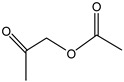	1-(acetyloxy)-2-Propanone	-	-	6.48	0.14	1.94	1.76
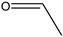	Acetaldehyde	2.55	3.03	7.47	0.64	-	-
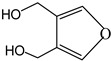	3,4-Furandimethanol	-	-	8.87	0.65	-	-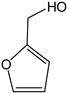	2-Furanmethanol	-	-	15.79	0.95	-	-
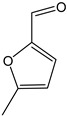	5-Methyl furfural	-	-	17.87	0.69	4.52	4.52
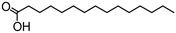	Hexadecanoic acid	-	-	25.99	0.27	-	-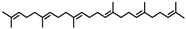	Squalene	-	-	31.04	0.17	-	-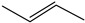	2-Butene	-	-	-	-	1.51	14.58
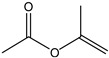	Isopropenyl acetate	-	-	-	-	1.63	11.97
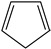	1,3-Cyclopentadiene	-	-	-	-	1.74	3.76
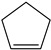	Cyclopentene	-	-	-	-	1.80	1.29
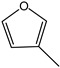	2-Methylfuran	-	-	-	-	1.87	0.66
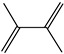	1,3-Butadiene	-	-	-	-	2.07	0.28
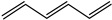	1,3,5-Hexatriene	-	-	-	-	2.2	1.95
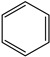	Benzene	-	-	-	-	2.37	1.67
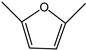	2,5-Dimethylfuran	-	-	-	-	2.78	1.39
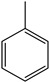	Toluene	-	-	-	-	3.79	2.27
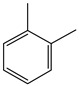	1,2-Dimethyl benzene	-	-	-	-	6.26	1.89
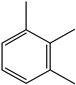	1,2,3-Trimethyl benzene	-	-	-	-	8.8	0.77
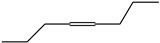	4-Octyne	-	-	-	-	11.54	0.28
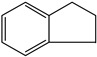	Indene	-	-	-	-	11.97	0.34
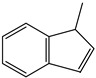	1-Methyl indene	-	-	-	-	17.18	0.96
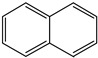	Naphthalene	-	-	-	-	18.09	0.32
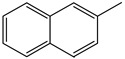	2-Methyl naphthalene	-	-	-	-	20.09	0.84
